# Deep Learning, Mining, and Collaborative Clustering to Identify Flexible Daily Activities Patterns

**DOI:** 10.3390/s22134803

**Published:** 2022-06-25

**Authors:** Viorica Rozina Chifu, Cristina Bianca Pop, Alexandru Miron Rancea, Andrei Morar, Tudor Cioara, Marcel Antal, Ionut Anghel

**Affiliations:** 1 Computer Science Department, Faculty of Automation and Computer Science, Technical University of Cluj-Napoca, 400114 Cluj-Napoca, Romania; viorica.chifu@cs.utcluj.ro (V.R.C.); cristina.pop@cs.utcluj.ro (C.B.P.); alexandru.rancea@cs.utcluj.ro (A.M.R.); marcel.antal@cs.utcluj.ro (M.A.); ionut.anghel@cs.utcluj.ro (I.A.); 2 Montran SRL, Alexandru Vaida Voevod 16, 400592 Cluj-Napoca, Romania; amorar@montran.com

**Keywords:** daily routine, activities patterns, daily activity detection, pattern mining, collaborative clustering, deep learning model

## Abstract

The monitoring of the daily life activities routine is beneficial, especially in old age. It can provide relevant information on the person’s health state and wellbeing and can help identify deviations that signal care deterioration or incidents that require intervention. Existing approaches consider the daily routine as a rather strict sequence of activities which is not usually the case. In this paper, we propose a solution to identify flexible daily routines of older adults considering variations related to the order of activities and activities timespan. It combines the Gap-BIDE algorithm with a collaborative clustering technique. The Gap-BIDE algorithm is used to identify the most common patterns of behavior considering the elements of variations in activities sequence and the period of the day (i.e., night, morning, afternoon, and evening) for increased pattern mining flexibility. K-means and Hierarchical Clustering Agglomerative algorithms are collaboratively used to address the time-related elements of variability in daily routine like activities timespan vectors. A prototype was developed to monitor and detect the daily living activities based on smartwatch data using a deep learning architecture and the InceptionTime model, for which the highest accuracy was obtained. The results obtained are showing that the proposed solution can successfully identify the routines considering the aspects of flexibility such as activity sequences, optional and compulsory activities, timespan, and start and end time. The best results were obtained for the collaborative clustering solution that considers flexibility aspects in routine identification, providing coverage of monitored data of 89.63%.

## 1. Introduction

According to the World Health Organization (WHO), it is expected that the number of older adults aged over 60 years old will double by 2050 [1]. In a society where life expectancy is constantly increasing, the implementation of sustainable solutions for the healthcare of older adults should have a high priority. Novel care solutions must be designed to allow older adults to stay longer at home in a familiar and comfortable environment, and to receive regular personalized health support, and follow-up from medical staff [2]. They decrease the costs of carrying individuals enabling the implementation of personalized guidance and recommendation services to improve general health and wellbeing.

In this context the monitoring of older adults in their homes through infrastructures based on sensors has proven to be a viable alternative to complement long-term care services provided by formal (medical staff, professional caregivers, etc.) or informal (family, friends, etc.) caregivers. The sensor-based monitoring of activities and follow-up are fundamental for supporting the relocation of care from hospital to home and the implementation of new evidence-based payment models in the healthcare system [3]. In this way, existing gaps concerning translational care can be addressed more efficiently, ensuring, at the same time, reductions in spending and improving care quality for populations with age and social risk factors [4].

Through a sensor-based monitoring infrastructure, real-time access can be obtained to data describing the daily living activities performed by a person as well as their health status. Such data is useful to identify a possible deterioration in the person’s health status or emergency situations, generate personalized interventions, but also alert caregivers in case of emergency situations [5]. Data can be fused, and machine learning techniques and technologies can be used to extract patterns and detect anomalies or situations that require intervention [6]. The daily living activities data of a person could be used to detect regular daily activities patterns (i.e., daily routine) as well as deviations from them [7]. The daily routine describes information on activity type, frequency, and sequence [8] and are key elements in transforming the information collected from sensors into useful observations through which the medical staff can draw conclusions regarding the change in adults’ health conditions. For example, the functional ability of older adults is an important element for tracking their health and well-being and it is reflected by the changes that can be registered in their daily routines [9].

In relation to the daily routine of older adults monitoring motion sensors has been widely used [10]. They have advantages related to the low cost and usability but are difficult to be used to capture the activities when the older adults are not living alone [11]. Nowadays, wearable devices such as smartwatches and activity trackers, monitor and estimate some basic activity levels related to physical activity or sleep quality [12]. Even though they are good candidates for tracking the daily routine and studies have shown their utility in monitoring activities [13] in the case of older adults, only a few solutions go beyond physical and sleep activities [14]. Moreover, the daily routine defined as frequent sequences of activities, with a similar timespan is not identified and exploited even though they may be very relevant in managing chronic diseases or dementia often encountered in the elderly population [15].

Several state-of-the-art studies [14,16,17,18] are addressing the detection of older adults’ daily routines. However, most approaches use small sequences of activities or even one type of activity and do not consider the entire day. Moreover, the daily routine is considered a rigid sequence of activities, but the daily routine may feature various flexibilities related to the activities performed, the time span, and the start and end times. The solution proposed in this paper enables the consideration of flexible aspects in daily routine detection allowing to better differentiate the normal and abnormal activity patterns. To the best of our knowledge, none of the state-of-the-art studies considers flexibility elements and the daily routine to be composed of subroutines specific to each period of the day. Addressing the daily routine at this granularity helps to better consider the timespan of activities and to have more refined control and flexibility in considering the activities detected more than once in the same day.

The paper is structured as follows: Section 2 reviews the state of the art and Section 3 highlights the paper’s aim and novel contributions beyond state-of-the-art. Section 4 presents the theoretical background and the proposed solution for detecting a person’s daily routine. Section 5 presents the experimental evaluation, while Section 6 presents conclusions and possible further developments. Table 1 describes the terms and technical abbreviations used throughout the paper.

## 2. Related Work

Identifying a person’s daily routine is an important issue because it allows us to detect changes that may occur in a person’s behavior, changes can be related to health problems. The literature approaches can be grouped into three main categories, namely, unsupervised approaches [14,16,17,18,19,20], supervised approaches [21,22,23,24,25], and statistical or model-based approaches [15,16,17,18]. All these state-of-the-art approaches define the daily routine of a person as a sequence of activities performed throughout the day. For identifying the daily routine, besides the order in which the activities are performed the following features are considered: the timespan associated with each activity, the time when the activity is performed, and the location and posture of the person performing the activity.

In [19], the authors propose an unsupervised learning method for detecting the daily routine of elders. The method combines the kernel K-Means algorithm with a novel nominal matrix factorization. The following steps are used: (i) representation of the trajectory data utilizing a behavior-aware flow graph, (ii) identification of the sub-flows representing behavioral patterns with kernel k-means algorithm, and (iii) identification of the daily routine using a nominal matrix factorization method. The daily routine is defined as a sequence of activities linked to the interval in which they are performed. In [16], the behavior routine of a person is extracted using an unsupervised approach based on the DBSCAN algorithm. The DBSCAN algorithm is applied to the historical data to cluster the activities based on the start time and the timespan. After eliminating the noise, the average start time and timespan of each activity are computed, and the daily routine is built.

Article [17] proposes an unsupervised approach for extracting the daily routine of elders and detecting deviations from it. The approach implies identifying the activities of daily living, daily routine modeling, and detecting the deviations from the daily routine. It is applied to data collected from sensors to generate fuzzy rules that establish a correlation between the appliances’ power signatures and the locations of the occupant during performing activities of daily living. The rules are used to identify the activities of daily living based on the appliance usage. The daily routine is modeled by extracting the features from data related to the appliance’s usage. A set of fuzzy rules are defined by mapping the values of the features to different activities of daily living levels, which are then used to detect deviations. In [14], the daily routine is extracted using the Partition Around Medoids algorithm for clustering vectors of sensor data and vectors of daily activities. The goal was to identify days with similar daily patterns and use them to extract the daily routine. As for similarity measurements, the Hamming distance is used when comparing sensors data and the Levenshtein distance is used when comparing vectors of daily activities, which may have different lengths due to the varying timespan of activities. Authors of [18] propose an unsupervised approach for extracting the user’s habit from the object and ambient sensors data. Based on the collected information, the activities are represented as Fourier series, where the timespans and sequence of the activities are the phases and amplitudes of the harmonics. These numerical representations are clustered in groups using the K-Means algorithm, and each group represents a category of patterns with similar behaviors. The habit is extracted by analyzing each pattern associated with different clusters.

Article [25] proposes a supervised approach for learning the daily routines of a person. The proposed approach is based on a three-layer self-organizing neural network model, namely the Spatiotemporal ADL Adaptive Resonance Theory. The first layer of the network contains information about the activities of daily living type, time, day, and space. The second layer encodes the association between the activity types and the spatiotemporal information, while the third layer encodes the sequential combinations of the spatiotemporal activities. The daily routine is extracted by considering the time, timespan, and spatial information as well as the type of the day (i.e., weekday or weekend). Article [24] uses three neural networks for activity recognition and abnormal behavior detection in the case of elders with dementia. The three neural networks are applied using a methodology consisting of four steps. First, a sliding window algorithm is applied to segment the raw data. Second, the most relevant features are extracted from the segmented data set. Third, the neural networks are trained to recognize the daily life activities and to learn the daily life behavioral routine. Finally, the trained model is used to identify abnormal behavior. Like [21], article [24] applies Long Short-Term Memory (LTSM), Convolutional Neural Network (CNN), and Autoencoder-CNN-LSTM to identify the daily routine and predict the deviation from it in the case of elders. Article [22] uses machine learning techniques to detect daily patterns. To select relevant contextual features and fine-tune the learning parameters, heuristics are used.

The authors of [26] propose an approach to building the daily mobility routine of an elder based on data collected with a low-cost monitoring infrastructure. The mobility profile of the elder is represented as a room-to-room state transition model, while the elders’ daily stay and room-to-room transition behavior is represented by a transition probability-based matrix. Each possible transition in the state transition model has a probability associated that represents the estimated probability of that transition. To estimate the probability of a person staying or navigating between the rooms of the house three types of probabilities are considered: the compute self-transition, the stay probability, and the compute transition probability. The transition probability matrix together with the concepts of global activity, inter-room activity, intra-room activity, and intra-room continuous stay, are used to build a model of the daily mobility behavior of a person. Article [27] proposes an approach for extracting the behavioral pattern of a person by considering the activity, activity timespan, the activity location, and posture of the person performing the activity. For identifying the behavioral pattern of a person, a statistical method is used, namely Hidden Markov models (HMM). Posture verification and the activity timespan were able to identify when a person’s behavior is normal and when it is not. The Viterbi algorithm was used to test the validity of the obtained model over a set of testing days. The approach presented in [28] learns the behavioral routine of an individual using an inverse reinforcement learning-based solution and by considering the Spatio-temporal information. For this, the sequential decision-making space of the resident in a smart home is modeled as a Markov Decision Process, and then it is used to learn the resident’s behavioral routine via relative entropy inverse reinforcement learning. Article [29] proposed a method for analyzing elderly daily behavior and deviations from it using motion and state-change sensor data. First, a probabilistic Spatio-temporal model is defined to represent the elder’s daily behavior. Then, anomalies, defined as being significant changes from the learned behavioral model, are detected using the cross-entropy measure. Ref. [30] develops a system that can learn the behavior of a person and detect behavioral changes, based on data about the performed daily activities collected from sensors. The novelty consists in combining a grey model with a Markovian model to extract the routine and detect abnormal situations.

Table 2 presents a comparative analysis between our approach and other state-of-the-art approaches from the research literature.

## 3. Aim and Research Contribution

**Figure 1:**
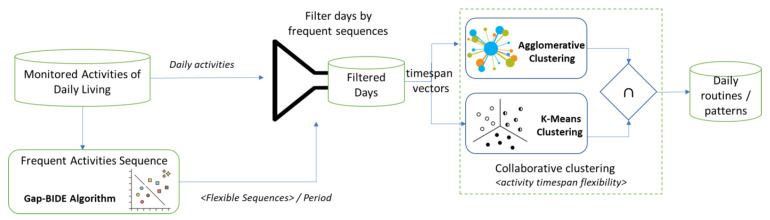
Daily routine identification considering flexibility aspects. Frequent activities are extracted with the Gap-BIDE algorithm while Collaborative Clustering is used to group and detect activity patterns. The identified clusters are represented in the graphs as geometric forms with different colors.

The paper aims to analyze sensors-based data acquired from a smartwatch device worn by an older adult to detect activities of daily living and frequent patterns of activities or routines considering several elements of flexibility. We consider the routine as a set of consecutive daily living activities of a person that repeat often [32] allowing variations in terms of the sequence of activities performed by the person and the average timespan of the activities considered. We consider activity sequences or sub-routines for each period of the day (i.e., night, morning, afternoon, and evening) and use the Gap-BIDE algorithm [33] to extract the most frequent ones (see Figure 1). We use cooperative clustering to consider flexible activities timespan in our routine. K-Means and Agglomerative Hierarchical Clustering algorithms are applied to group the most frequent behavioral patterns considering the timespan vectors. Finally, the days containing the most common patterns and for which the average timespan of activities is equal to the vector corresponding to the centroids of the clusters to which those patterns belong are selected.

As reported in the Section 2 most of the existing research works focus on extracting the individual’s daily routine which is seen as a rather rigid sequence of daily life activities. Our proposed solution goes beyond the existing state of the art by considering daily routines featuring a certain degree of flexibility. We introduce slight variations in daily routine in terms of the sequence of the activities performed and the average timespan of activities. In the state-of-the-art literature, the timespan of specific daily life activity is computed based on the timespans of that activity concerning the whole day. We determine the daily activity timespan considering the part of the day when the activity takes place. For example, the timespan of daily living activity can differ in the morning compared with the afternoon and still be considered part of the routine. In our case, the activity timespan corresponds to a range of values established using two thresholds computed based on the clustering results and activity type. We distinguish among instant, short, medium, and long activities that are more appropriate than using a simple average of the timespans. The time interval in which an activity takes place is determined as the minimum between the start times of that activity and the maximum between the end times of that activity also considering the part of the day (i.e., morning, afternoon, evening, and night). This is a more realistic approach for computing the time interval associated with each activity rather than having fixed start and end times. In our definition of daily routine, we have considered mandatory activities (i.e., activities that are performed regularly every day), optional activities (activities that may be performed or may be missing in a day), and alternative variants of activities (groups of activities that may differ in the number of performed activities, the activities name, the time interval in which an activity is performed or the activity duration). This reflects better how people carry out their daily activities. Even if in a day some optional activities are missing, or instead of performing an activity, a group of activities is performed (e.g., instead of performing showering in the morning, showering followed by toileting is performed in the afternoon), that person still may follow a daily routine and our solution can detect it. Finally, we split the daily routine into four sub-routines, one for each part of the day (i.e., night, morning, afternoon, and evening). Composing the daily routine of a person from the daily routines corresponding to each part of the day provides good results for the case in which the average duration of an activity is different depending on the part of the day in which the activity is performed.

This paper’s novel contributions are summarized below:

- Definition of flexible daily routines incorporating elements of slight variation as opposed to state-of-the-art approaches which consider the routine as a rather rigid sequence of activities. The elements of variation are related to the sequence of activities in a routine as well as the timespan of the activities.
- Identification of frequent behavior patterns using the Gap-BIDE algorithm and considering the elements of variations and sub-routines in activities sequence (i.e., night, morning, afternoon, and evening) for increased pattern mining flexibility.
- Address the time-related elements of variability in daily routine by using K-means algorithms and Hierarchical Clustering Agglomerative algorithms onto activities timespan vectors having as similarity metrics the dynamic time warping and Manhattan distance.
- A prototype to monitor and detect the daily living activities based on smartwatch data using a deep learning architecture and the InceptionTime model for which the highest accuracy was achieved.

## 4. Flexible Daily Routines

When defining the daily routine of older adults is important to have flexibility because the routine may vary slightly from day to day [34]. Having some level of flexibility could help to identify more precisely the deviations from the daily routine of elders. Also, it is important to make a distinction between the morning routine, afternoon routine, evening routine, and night routine. In the case of older adults, the morning is the most active time, the afternoon is dedicated most of the time to leisure activities, while the evening is dedicated to dinner or enjoyable activities, etc.

We leverage these assumptions to define the concept of a flexible routine. It incorporates elements of slight variation related to the sequence of activities, start time and end time of each activity, and activity timespan. In this case, the daily routine of a person is modeled as a sequence of four sub-routines corresponding to each period of the day (i.e., night, morning, afternoon, and evening).

(1) $$dailyRoutine=\{routine_{period}|\;period\in \left\{night,\;morning,\;afternoon,\;evening\right\}\;\}$$

where the sub-routine corresponds to a period of the day, $routine_{period}$ is defined as a sequence of activities, each activity having associated a timespan, the start and end time, and a variable showing if the activity is mandatory or not.

(2) $$routine_{period}=\left\{\left(a_{i},\;timespan,\;t_{start},t_{end},\;optional\right),\;\left(a_{i+1},\;\ldots \right)\ldots \left(a_{n},\;\ldots \right)\right\}\;$$

The optional variable specifies if the activity is mandatory (i.e., it is an activity that is performed regularly every day), optional (i.e., it is an activity that may be performed or may be missing in a day) or if it is part of an alternative variant of activities (groups of activities that may differ in the number of performed activities, the activities name, the time interval in which an activity is performed or the activity timespan).

The *timespan* of an activity is computed according to the period of the day in which the activity is performed (i.e., the timespan of Spare time/TV activity in the morning can be different from the timespan of Spare time/TV activity in the afternoon) and depends on the category to which the activity belongs:

(3) $$timespan\;\left(a_{i},period\right)=\frac{\sum_{a_{i}\in \;days}^{a_{i}\in \;period}timespan\;\left(a_{i}\right)}{count\left(a_{i}\right)}$$

The start and end times of the activities in a specific period of the day are computed as follows:

(4) $$t_{start}\left(a_{i},period\right)=\underset{a_{i}\;\in \;days}{\min}t_{start}\left(a_{i},\;period\right)$$

(5) $$t_{end}\left(a_{i},period\right)=\underset{a_{i}\;\in \;days}{\max}t_{end}\left(a_{i},\;period\right)$$

where *days* represents the number of days from which the routine is extracted.

We use the Gap-BIDE algorithm to identify the frequent activity sequences in each period of the day, then we are filtering those days having frequent sequences in more periods, and finally, we employ a collaborative clustering solution to consider the activity timespan flexibility in routing identification.

### 4.1. Frequent Activities Sequences

**Figure 2:**
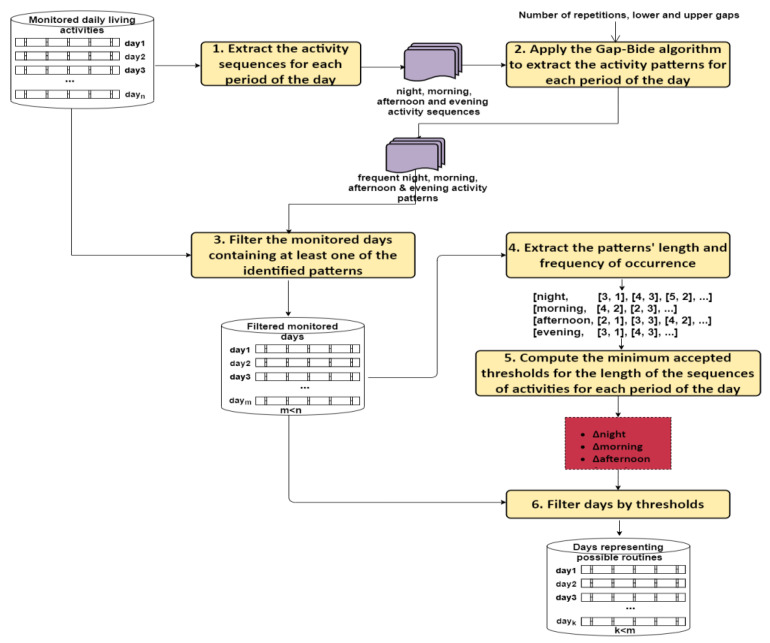
Frequent sequence identification using Gap-BIDE algorithm. Six steps are defined for extracting days that could represent routines or patterns.

We define the frequent behavior pattern as the sequences of consecutive activities of daily living that are often repeated on various days. To identify such activity sequences that are frequent we have used the Gap-BIDE algorithm [33] while for identifying the days containing such patterns, we have used filtering techniques (see Figure 2).

The daily routine is composed of sub-routines corresponding to each period of the day (i.e., night, morning, afternoon, and evening), thus we divided the activities performed in each day according to the period of the day, in which they are monitored. The periods of the day are defined in Table 3.

As a result of the activities spiting, we obtain sets of consecutive activities that are strongly connected to each other. As some of the specific activities of a person are performed more than once during a day, the splitting based on the periods of the day allows the observation of the cause-effect phenomenon for short series of activities. For example, we can detect correlations between a longer and several additional activities of shorter timespan caused by it in the next period.

The sequences of activities per period are provided as inputs to the Gap-BIDE algorithm to identify the most frequent behavioral patterns. We have used the Gap-BIDE algorithm because it allows specifying restrictions on the distance between two activities, part of a behavioral pattern. This offers a degree of flexibility for identifying non-rigid daily routines. It allows the analysis of frequent sequences composed of activities that have a high degree of connection between them (e.g., the *sleeping* activity will cause, in most cases, in a short time the appearance of one of the activities: *toileting, showering, or personal hygiene*). Also, it helps to identify frequent sequences composed of repetitive activities that may occur at different times of the day with no causal link between them. For example, *Sleeping* and *Eating* are frequent activities found in most of the days, but with other activities interleaved between them. Finally, the Gap-BIDE algorithm can determine activity sequences of a longer length by combining smaller length sequences. Many shorter activity sequences are often included in other activity sequences of longer length that reproduce accurately a behavioral pattern.

The activity sequences provided as input could have different lengths. Thus, we have provided the following input parameters to the algorithm: the minimum number of repetitions of an activities sequence to be considered frequent and the lower and upper ends of the potential gap interval between activities. The last two parameters define the number of consecutive activities that may separate two activities that are part of the same pattern. For example, a gap in the range [2, 4] specifies that all activities in a frequency sequence must contain at least 2 and at most 4 activities at a distance from each other (which are not part of that frequent sequence. In our case, we have run the algorithm with a value of zero on both parameters so that all results will contain sequences with consecutive activities and no gaps.

The patterns Identified with Gap-BIDE are used to filter from the set of all days only those days that contain these patterns also considering the period of the day.

We filter those days that contain the largest number of frequently identified patterns for at least 3 periods of the day. The sequence of activities should be carried out in the same order (without other interspersed activities and without permutations) as identified by the Gap-BIDE algorithm. The filtered set may contain days that are composed of a larger number of activities than the average number on all the other days. We filter them according to the four periods of a day considered. We extract from all days, the distinct lengths of activities sequences and the frequency of the occurrence of those lengths:

(6) $$<period,\;\left(lenght_{1},\;frequence_{lenght_{1}}\right),\ldots ,\left(lenght_{n},\;frequence_{lenght_{n}}\right)>$$

where: *length* is the length of some sequence of activities that are part of the filtered days and correspond to a period, $frequence_{lenght}$ is the frequency of occurrence of that length, *n* is the number of distinct lengths corresponding to the sequences of activities. Based on the frequency of occurrence of a length, we compute a minimum threshold for the activity sequences of that length to be accepted.

**Figure 3:**
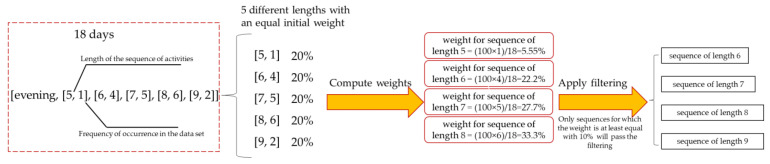
Filtering the sequences of activities based on the frequency of occurrence. Example for five different length sequences.

To illustrate this filtering process, we consider the example from Figure 3, where we have 5 different lengths for the evening sequences of activities: the sequence of length 5 appears once in the set of days, the sequence of length 6 appears four times in the set of days, etc. Initially, each sequence of activities corresponding to evening is assigned an equal weight of 20% of the total set. As certain sequences of marginal lengths (sequences of length 5 or length 9) are usually less common, it can be said that any sequence will pass the filter if the length of that sequence is met by at least 10% (half of what was initially allocated for each length) of cases. Consequently, in our case will pass the filtering, only the sequences of lengths 6,7, 8, and 9.

For a day to pass the filtering, all sequences of activities identified that correspond to a minimum of 3 of four periods of the day must reach the minimum threshold computed. It eliminates not only those sequences whose lengths are too small/too long (which have lengths at maximum distances from the arithmetic mean of the lengths of all sequences), but also eliminates any set of activities with a low length-frequency value (even if the length of that sequence is close to the arithmetic mean of the sequence lengths for that day).

### 4.2. Activity Time Related Variability

The days containing frequent sequences of activities filtered are further processed to allow the consideration of additional flexibility related to the timespan of each activity. We use a collaborative clustering technique by using parallel K-Means and Agglomerative Clustering algorithms (see Algorithm 1).
**Algorithm 1: Collaborative Clustering****Inputs:**dailyTimespanVectors—timespan of activities for all days and all periods of the dataset**Outputs:**dailyRoutine—activity sequence representing the baseline.**Begin**1*filtredDays_K-means_* = **K-Means Clustering**(dailyTimespanVectors)2*filtredDays_AG_* = **Agglomerative Clustering**(dailyTimespanVectors)3*filtredDays* = **Intersect***(filtredDays_K-means_, filtredDays_AG_)*4*dailyRoutine* = **Extract**(*filtredDays)***End**

They are used to cluster the timespan vectors corresponding to the sequences of activities of the days identified as featuring frequent patterns. The length of a vector is equal to the number of distinct activities while the timespan is computed as an average:

(7) $$timespan\;\left(a_{i},period\right)=AVG\;{\left(timespan\;\left(a_{i},period\right)\right)}_{days}\;$$

If, in a sequence, an activity is performed several times, then the timespan of that activity is considered in the average.

In the case of the K-Means algorithm, the set of clusters’ centroids are determined. For each sequence of activities performed, the index of the cluster to which it belongs is determined using Dynamic Time Warping as similarity metrics. Finally, we compare the timespan vector corresponding to that sequence of activities with the centroid of the cluster of which the sequence is part. For each activity from the sequence, we compute a ratio between the timespan of that activity and the timespan of the same activity in the centroid as:

(8) $$ratio_{a_{i}}=\frac{timespan\left(a_{i},period\right)}{timespan{\left(a_{i},period\right)}_{centroid}}$$

The range of values in which the variation of the timespan of the activity is allowed using the formula below:

(9) $$threshold_{L,H}=timespan\left(centroid\left(a_{i}\right)\right)\;\pm \alpha \times timespan\left(centroid\left(a_{i}\right)\right)$$

where *α* represents a percentage value that can be adjusted based on the experimental results. The two thresholds are used to establish the range of allowed values for the timespan of the activity:

(10) $$\{\begin{array}{c} if\;ratio_{a_{i}\;}<1,\;then\;timespan\left(a_{i},period\right)\in \left[threshold_{L,\;}timespan\left(centroid\left(a_{i}\right)\right)\right]\;\\ if\;ratio_{a_{i}\;}<1,\;then\;timespan\left(a_{i},period\right)\in \left[timespan\left(centroid\left(a_{i}\right)\right),\;threshold_{H}\right] \end{array}$$

A sequence of activities passes this filtering if each of its activities is in the determined range.

**Figure 4:**
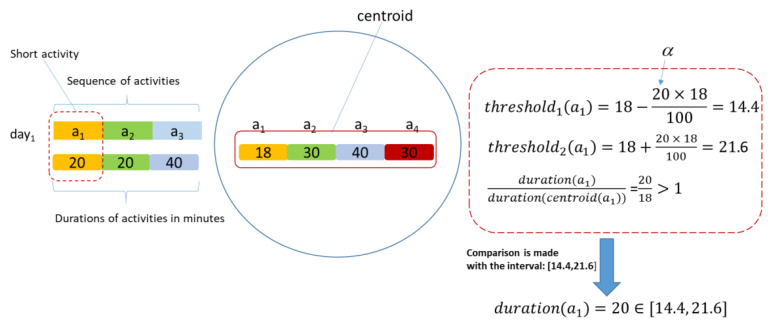
Timespan of an activity and clusters centroids. Example for computing the timespan for activity *a*_1_.

Figure 4 illustrates the steps followed to establish if the timespan of activity *a*_1_ is in the range computed based on the timespan of the same activity in the cluster centroid and depending on the type of activity.

The Agglomerative Clustering algorithm also takes as input the list of vectors of timespans corresponding to sequences of activities. It is used to extract only the information related to the cluster to which each sequence of activities belongs. A day will pass the filtering if each of the sequences of activities corresponding to each period of the day is part of a representative cluster (i.e., a cluster for each the number of items is above the minimum acceptance threshold).

The results of applying the two clustering algorithms are used to extract the daily routine. More exactly, we select only the days that are part of both days’ sets (i.e., days obtained as a result of the intersection between the two sets of days) returned by the two clustering algorithms as a result of the filtering process and analyze them. The daily routine comprises a basic sequence of activities (which corresponds to the most frequent sequences of activities) that are part of a person’s daily schedule, along with the normal intervals in which each activity is performed and the corresponding average time span. Also, besides this basic sequence of activities, the routine does not exclude the interleaving of other activities. We may have certain activities that are optional at a certain time of the day but may be mandatory or not at another time of the day. As a result, we can identify daily routines with elements of variation to a basic sequence (i.e., missing or more activities).

## 5. Experimental Results

In this section, we present the deep learning infrastructure used the detect the activities of daily living out of monitored data and the evaluation results on the detection of daily routing considering the flexibility features.

### 5.1. Activity of Daily Living Detection

**Figure 5:**
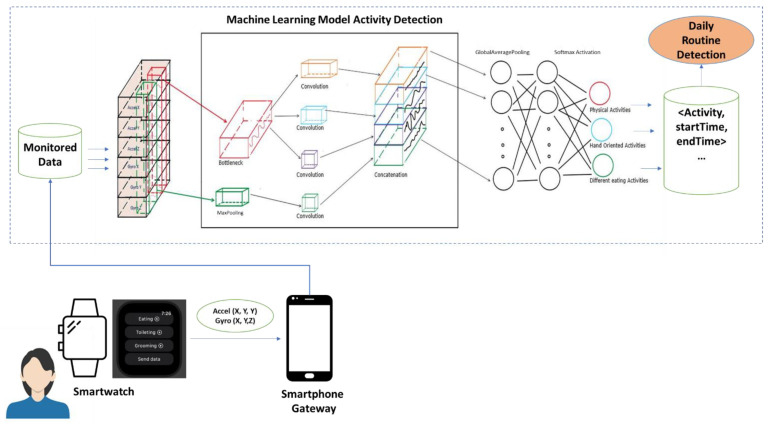
Architecture for data gathering and activity patterns detection. A smartwatch is used for collecting raw data that is ushed through a gateway (e.g., smartphone) in a cloud database.

To test and evaluate the proposed solution we have first constructed a prototype to monitor and detect the daily living activities so they can be then used for daily routine detection. A smartwatch application was developed to record data from accelerometer and gyroscope sensors and to label the data with the type of activity done (see Figure 5).

In general, smartwatches are successfully detecting sleeping and times outside of physical activities. So, for these times, we use the activity detection software available on the smartwatch. To determine other types of activities such as eating (e.g., Breakfast, Lunch, Dinner, Snack), toileting, personal hygiene, and spare time/TV a machine learning model was used. The smartphone is used as a gateway for collecting the data from the smartwatch sensors and forwarding it to the cloud. Each recorded data is composed of 6 values accelerometer (*X*, *Y*, *Z*) and gyroscope (*X*, *Y*, *Z*) the sampling rate being 100 Hz which provides the option of down sampling if needed.

WISDM Smartphone and Smartwatch Activity and Biometrics Dataset [35] is used to train the machine learning model. This dataset is composed of data from 51 users that were required to do certain activities for 3 min while recording the accelerometer and gyroscope data. The machine learning model uses a multi-step categorization of the activities. First, the general categories of activities that could represent different types of activities are identified: Physical Activities, Hand Oriented Activities, and Eating Activities. Afterward, specific models run to discriminate among activities in the same category. In this way, the size of the data set is decreased by 80% from ~51,840,000 values/day to ~10,368,000 values/day and then it is split into 10 s intervals using a 30–50% overlap.

To determine the most suitable deep learning architecture for our case three different types of models were tested [36]: InceptionTime, DeepConvolutionLSTM, and ResNet. Using the mcfly framework [36], we were able to generate about 15 different models for each type of daily living activity we are aiming to detect. The highest accuracy model has been achieved InceptionTime architecture. It is composed of a Bottleneck and MaxPool layers, 3 Convolutional layers, a Concatenation layer, a GlobalAveragePooling layer, and a Dense layer. The Bottleneck layer is constructed by 1 × 1 convolutions with the number of outputs lower than inputs. By reducing the number of features, recurrently, the model’s computing necessities are decreasing. MaxPooling layer uses a convolution process where a filter calculates the highest value in each patch. The output of this MaxPooling layer is a summarized version of the previous layer containing the maximum value in each region. It helps generalize the variations, making a more robust model. Convolutional layers are also found in different sizes. A concatenation layer is also used for merging a list of inputs, in our case the output of the convolutional layers. The GlobalAveragePooling layer is used to decrease the spatial dimensions to just a 1 × 1 × *C*, where *C* is the number of channels. It enforces a correlation between each feature map to one daily activity category of the classification to avoid overfitting. The GlobalAveragePooling layer is followed by the Dense layer that uses a softmax activation function to determine the predicted daily activity class. The softmax activation function transforms the vector of values to a probability distribution with values between 0 and 1 that sum up to 1 and represent the highest probability category.

**Figure 6:**
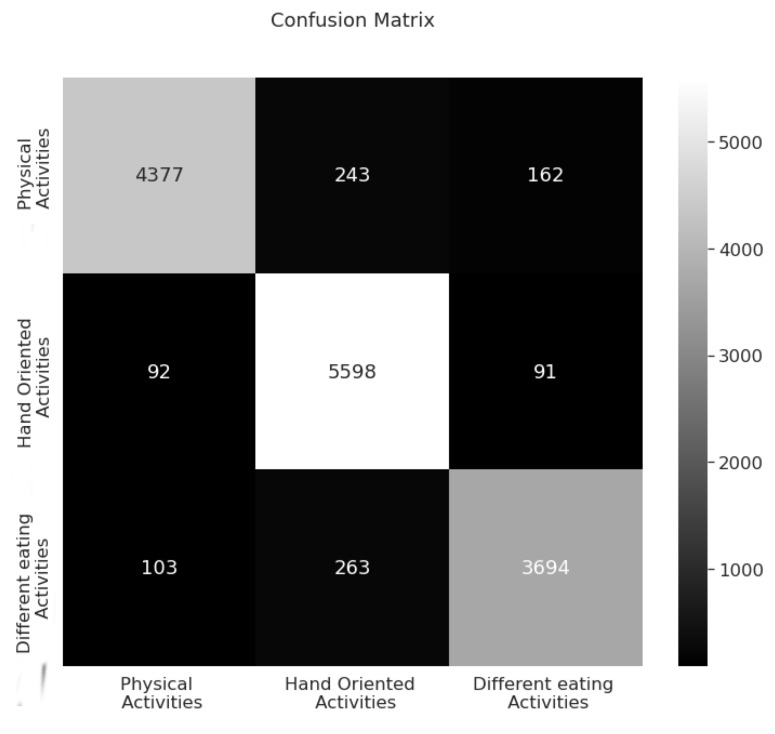
The confusion matrix for activity detection. The better the model, the values outside the main diagonal are lower compared to the ones on the main diagonal.

The confusion matrix from Figure 6 is used to represent the prediction results of the proposed activities of the daily living classifier. Each row represents the actual activities classes and the columns represent the predicted classes. Each value in this matrix represents the count of correct and incorrect results of the classification process. The number of predictions of a class is placed into the expected class row and the predicted class column. The main diagonal represents the correct answers, and all the other values are incorrect predictions. For example Hand Oriented Activities category, from a total number of 5781 predictions, 5598 are correct and 183 are wrong.

**Figure 7:**
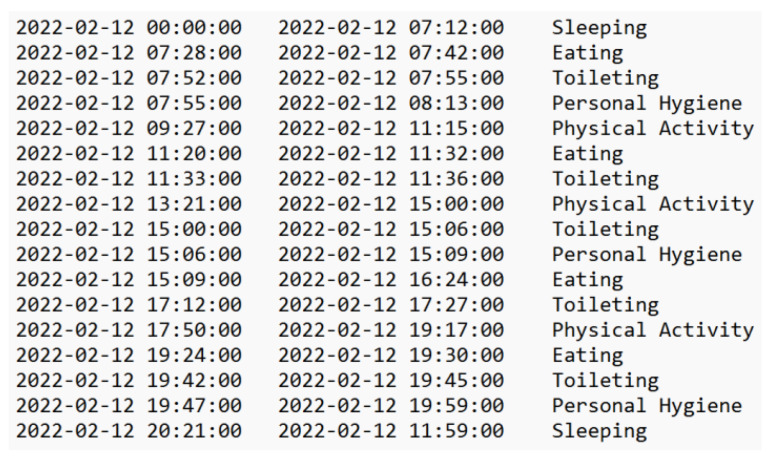
Daily activities detected for a person in a day (24 h).

Using the model presented above we can generate the Activity file which will be used as input for the person’s daily routine detection. It has the following structure on each line: startTime, finishTime, and activityType. Figure 7 provides a sample of the data set. The fragment contains information about the activities performed by a person in a day.

### 5.2. Daily Routine Detection

**Figure 8:**
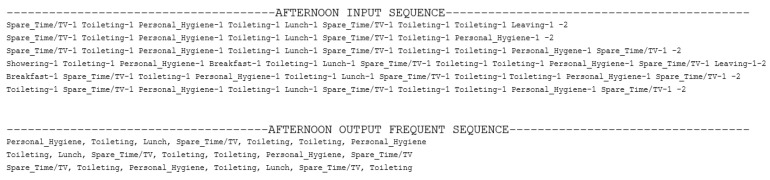
Frequent patterns (most frequent sequences of activities) for the afternoon identified by the Gab-BIDE algorithm.

To detect the most frequent behavioral patterns or the daily routines, we apply the proposed technique to 70% of the data. The days are extracted and feature a set of activities with the interval in which they are performed. The activities are grouped in sequences corresponding to the main periods of the day (i.e., Night, Morning, Afternoon, Evening) and are provided as inputs for the Gab-BIDE algorithm. Figure 8 presents the most frequent patterns identified by Gap-BIDE for the Afternoon.

These results are then used in the subsequent filtering steps to select the days that will be provided as input to the clustering algorithms. We apply the K-Means algorithm using the Silhouette score to assess the similarity among frequent patterns of the same cluster and the difference between frequent patterns in the different clusters:

(11) $$s\left(i\right)=\frac{b\left(i\right)-a\left(i\right)}{Max\left\{a\left(i\right),\;b\left(i\right)\right\}}$$

where: a(i) is the average distance between the frequent daily activities pattern *i* and the other frequent patterns in the same cluster $C_{i}$, and b(i) is the smallest average distance between the frequent pattern *i* and the frequent patterns in the other clusters, of which *i* is not a part. The values for *s*(*i*) are in the range [–1, 1]. A value close to 1 indicates that the items are well separated, the frequent daily activities patterns in the same cluster having a high degree of similarity, while the frequent patterns in different clusters have a low degree of similarity/high degree of differentiation. A negative score indicates incorrect grouping and usually occurs because of using too many clusters (data interleaving).

The Davies-Bouldin Index was then used to determine the intra-cluster similarity and inter-cluster differences:

(12) $$DB=\frac{1}{c}\sum_{i=1}^{c}Max_{i\neq j}\left\{\frac{d(X_{i})+d(X_{j})}{d\left(c_{i},c_{j}\right)}\right\}$$

where *c* represents the number of clusters; *d*(*X_i_*) and *d*(*X_j_*) are the distances between all frequent daily activity patterns in the cluster *X_i_*, respectively *X_j_*, and the centroids of those clusters, and *d* (*c_i_*, *c_j_*) is the distance between the centroids of the two clusters, *c_i_* and *c_j_*. The lower the value of the Davies-Bouldin Index, the better solutions are obtained. By analyzing the Silhouette score and Davies-Bouldin Index we determine the optimum number of clusters in the case of our daily activities data set. We use Silhouette analysis to determine the number of clusters for featuring: (a) a high average silhouette score, (b) cluster items with scores above the average silhouette, and (c) similar silhouettes in size.

**Figure 9:**
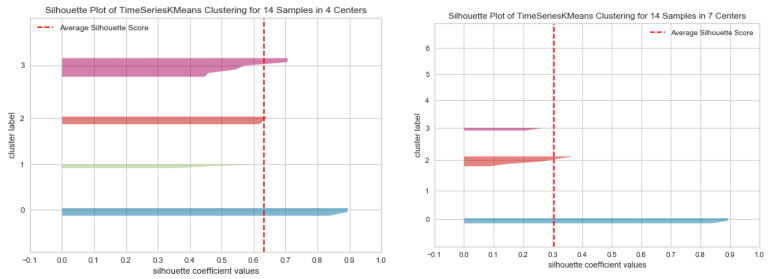
Silhouette analysis for K-Means clustering on time span vectors with k = 4 (**left**) and k = 7 (**right**). Cluster labels are colored differently. The average Silhouette score is shown with the dotted red line.

**Figure 10:**
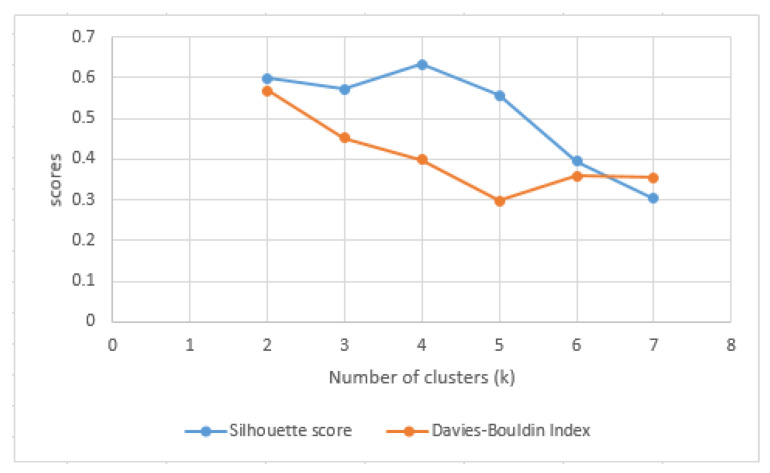
Silhouette score (blue) and Davies-Bouldin index (orange) relative to the number of clusters.

We have varied the number of clusters *k* in the range [2, *n*/2] where n represents the maximum number of items (i.e., time span vectors corresponding to the most frequent sequence of activities performed in a specific interval in the day). Figure 9 illustrates the Silhouette plots for different values of *k* in the range [2, 7], while Figure 10 presents the Silhouette score and Davies-Bouldin Index values relative to the number of clusters.

The value of 4 for the number of clusters is the optimal one, because even if we have clusters with the Silhouette score below the average, that average value is higher than for *k* = 2, 3, 5, 6, 7 and the Davies-Boulding index value is low (see Figure 10).

**Figure 11:**
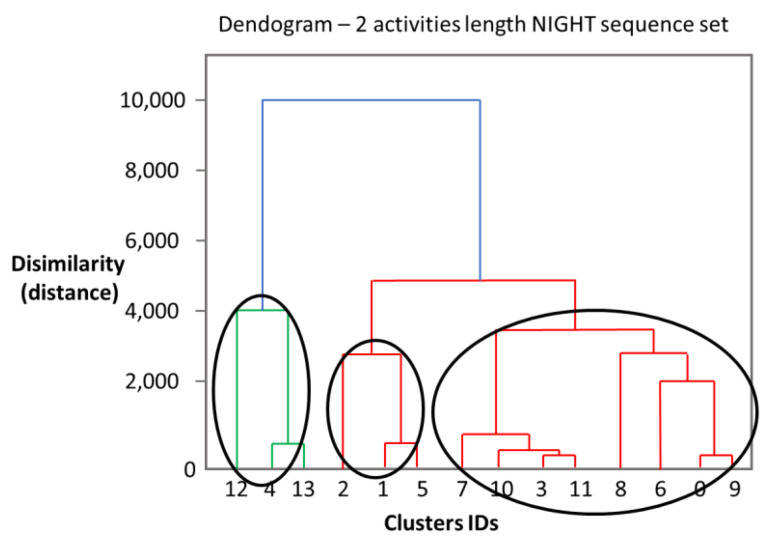
Dendrogram obtained when running the Agglomerative Clustering algorithm for Night sequences. On the *X*-axis the sequence ids are represented, and on the *Y*-axis the dissimilarity distance. Red, blue, and green colors are used to different clusters of activities created.

To determine the optimal number of clusters of daily activity patterns while using hierarchical clustering, we have used a dendrogram and the Dunn and Davies-Boulding indexes. A dendrogram is a tree chart representation that illustrates the process of cluster creation and merging. In a dendrogram, all possible variants of clusters are exemplified, together with the differences between any chosen groups (highlighted by means of the vertical distance between any 2 consecutive nodes). Nodes mean the union of two data sets, and the vertical distance between nodes represents the difference between the two groups. The greater the vertical distance is, the more different the two groups are. The Dunn index is defined as:

(13) Dunn Index=min1≤i≤c{min1≤j≤c, j≠i{δ(Xi, Xj){∆(Xk)}1≤k≤cmax}}

where $\delta \left(X_{i},\;X_{j}\right)$ is the inter-cluster distance (i.e., the distance between the cluster *X_i_* and the cluster *X_j_*), and $∆\left(X_{k}\right)$ is the intra-cluster distance of cluster $X_{k}$ (i.e., the distance within the cluster $X_{k}$). The optimum number of clusters is the number corresponding to maximized *Dunn Index*. Figure 11 shows the dendrogram resulting after running the Agglomerative Hierarchical Clustering algorithm on vectors of time span corresponding to the activity sequences that take place at Night (all activity sequences that take place at night are composed of two 2 activities).

As it can be noticed the optimal number of clusters for our case is 3. For a smaller number of clusters, the distance between clusters increases a lot (i.e., up to 6000). For a higher number of clusters, we have clusters with a single element. For example, if we have 4 clusters, the sequence with the id 12 would remain distributed in its own cluster with a single element. The calculated Dunn and Davies-Bouldin indexes are shown in Table 4 for varying the number of clusters in the range [2, 6]. We observe that the best values for the Dunn Index (i.e., when grouping vectors of time span corresponding to the sequences of activities performed during the night) are obtained when using 3 or 4 clusters. The two values are relatively close. The Davies-Bouldin index decreases for higher values of the number of clusters (i.e., 5, 6 clusters), because it also considers the clusters with a single element.

**Figure 12:**
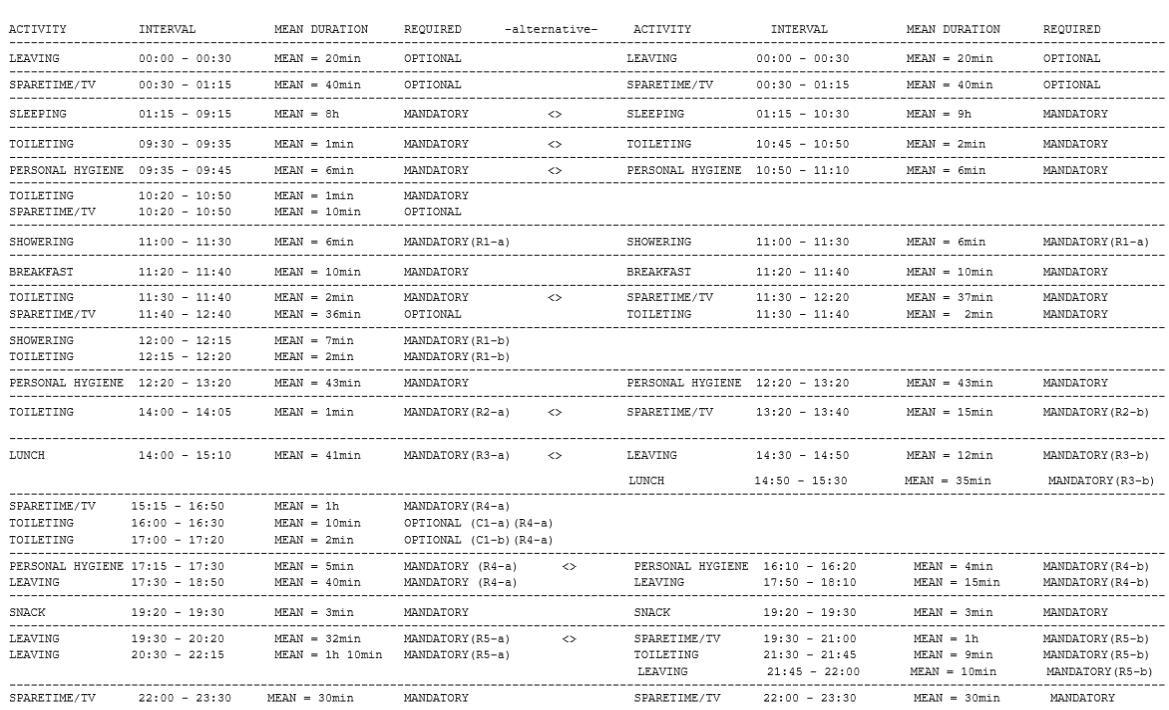
Example of flexible daily routine identified for a person for a full day (24 h).

Based on the results obtained with the two clustering algorithms we finally determine the daily routine of a person (see Figure 12). The routine is composed of the activities carried out daily by the person. It contains information about the activity, the time interval in which the activity is detected, the average timespan, and a flag, Required, that specifies if the activity is mandatory or optional. Also, the routine is flexible, with the same activity being carried out at different intervals of the day and with different timespans. For example, in Figure 12, the *Sleeping* activity can take place either in the interval [01:15 AM–9:15 AM] or [01:15 AM–10:30 AM]. In the first case, the time span of the *Sleep* activity is 8 h, while in the second case around 9 h. Entries proposing alternative variants that differ in the number and type of activities in an interval are marked with the symbol “<>”. For example, we can have in the routine the Toileting activity to be performed between 11:30 and 11:40 or the same time Spare time/TV activity can be performed between 11:30 and 12:40. These variants are accepted as being part of daily routine. 

## 6. Discussion and Conclusions

In this paper, we have proposed a solution to identify the activities of daily living and routines of a person with certain flexibility considering variations related to the sequence of activities (e.g., gaps in sequence) and activities timespan. To meet our goals, we have developed a solution to monitor and identify daily living activities, using deep learning models and data from smartwatches. The identified daily activities are then fed to the Gap-BIDE algorithm to filter those days that contain the most frequent patterns considering potential missing activities in sequence as elements of flexibility. To enrich the daily routine with information about the activities’ timespans and to allow activities time-related variability, a collaborative clustering technique was employed using in parallel to the K-Means and Agglomerative Clustering algorithms. The results are promising the Gap-BIDE algorithm is effective in detecting frequent patterns of activities even with slight gaps in activity sequences, scaling well concerning the dimension of the data set. Also, the collaborative clustering technique successfully addresses the timespan variation of the activities in the routine since it combines the results provided by the individual clustering algorithms which increase the clustering quality.

To analyze the results obtained and to identify the optimal approach for combining the results of the clustering algorithms, we have determined the coverage of the determined routine over the monitoring days. The coverage metric determines the number of days from the entire monitoring history that is compatible and follows the determined routine. Out of the days used to extract the routine, we have selected the ones that contain patterns from the daily routine, and at least half of their activities are also part of the frequent patterns. We have filtered the days that contain activities for which the following conditions are satisfied. The frequency and timespan of the activity are in the range computed for the routine, and the time intervals in which the activity takes place overlap the intervals for that activity in the routine. Since the activity can be performed several times in a day but in different periods, for time intervals and timespan, also the period was considered in filtering. Table 5 presents the values of the coverage metric computed for several clustering configurations.

Individually K-Means algorithm provides a lower routine coverage value compared to Agglomerative Clustering. The reason is that the K-Means Clustering algorithm is considerably more restrictive since it outputs clusters of frequent activity sequences with timespans close to the ones corresponding to the clusters’ centroids. Thus, a frequent sequence with infrequent timespans may be distributed in a cluster only because it was not close enough to another centroid. Consequently, the dimension of the cluster to which the frequent sequence is assigned increases, and the centroid can change. After performing the final filtering, it is possible to have some frequent sequences with frequent timespans matching the routine eliminated from the cluster leading to a lower coverage metric value. The Agglomerative Clustering algorithm, on the other hand, provides better coverage values since it groups the frequent sequences of activities based on their timespan similarity; the sequences of activities with infrequent timespans will be placed in clusters with few elements which will be discarded after the filtering process, therefore only the sequences that are slightly different from one another will remain.

The computational complexity of the clustering algorithms was analyzed by varying the number of clusters while considering the impact on the algorithms’ execution time.

The results reported in Table 6 show that the impact on the execution time is minimum while the best execution time is for k = 4 clusters which is the optimum number of clusters as generated by dendrogram analysis and respectively by the Silhouette score. Also, we have analyzed the influence of the data set size on the clustering execution time. We have gradually increased the size of the data set used to extract the daily living routine considering the optimum numbers of clusters. Table 7 results show that the impact on the execution time is minimum.

Finally, we have analyzed the impact of different parameters on the performance of the deep learning models used to identify the individual daily living activities (see Table 8). Deep learning model accuracy improvement in daily living activity detection can be achieved by using a higher amount of input data and by increasing the number of epochs in training. The best results are achieved for a window size of 600 data samples from sensors each at 30 s, and after this value, no further improvements can be detected. The stride value determines the step between two consecutive samples. A lower value of this parameter offers a bigger test set but can also introduce overfitting.

Our solution novelty is flexibility given by the slight variations related to the sequence of activities performed by a person and the average timespan of the activities composing the routine. Collaborative clustering offers the best solution for considering flexibility in routine identification. Using the union operator in collaborative clustering allows the identification of the days with frequent patterns that may have been eliminated by the K-Means Clustering Algorithm but have a duration vector close to a significant cluster provided by the Agglomerative Clustering Algorithm.

In future work, we plan to refine the clustering approach by implementing an ensemble clustering technique that aims to run multiple clustering algorithms in parallel and apply a consensus algorithm that aggregates the individual clustering results in a final one. Moreover, the differentiation between the routines of weekdays and weekends combined with the incorporation of additional features such as the location and the frequency of appearance of each activity would provide more accurate insights into the daily life activities of older adults. Finally, to close the loop, we intend to integrate the proposed solution into a platform for detecting anomalies (i.e., deviations from the identified routine) in the daily life activities of older adults.

## Figures and Tables

**Table 1:** Abbreviations and letter symbols.

Abbreviation	Unit or Term
$a_{i}$	Activity *i*.
$frequence_{lenght}$	frequency of occurrence of a length of activity
$ratio_{a_{i}}$	Ratio between the timespan of an activity and the timespan of the same activity in the cluster centroid
$routine_{period}$	Sub routine corresponding to a period of the day
$threshold_{L,H}$	Low and High thresholds for the variation of the timespan of an activity
$t_{end}$	end time of the activities in a specific period of the day
$t_{start}$	start time of the activities in a specific period of the day
$∆\left(X_{k}\right)$	distance within the cluster $X_{k}$
ADL	Activities of Daily Living
*c*	number of clusters
CNN	Convolutional Neural Network
*d*(*c_i_*, *c_j_*)	distance between the centroids of the two clusters, *c_i_* and *c_j_*
*d*(*X_i_*)/*d*(*X_j_*)	the distances between all frequent daily activity patterns in the cluster *X_i_*, respectively *X_j_*, and the centroids of those clusters
*days*	number of days from which the routine is extracted
DBSCAN	Density-based spatial clustering of applications with noise
*filtredDays*	Set of days containing frequent sequence of activities
HMM	Hidden Markov models
*length*	the length of some sequence of activities
LTSM	Long Short-Term Memory
*timespan*	Average duration of an activity.
WHO	World Health Organization
α	Percentage value that can be adjusted based on the experimental results
$C_{i}$	Cluster of activities *i*
DB	Davies-Bouldin Index
Dunn Index	Dunn index
a(i)	the average distance between the frequent daily activities pattern *i* and the other frequent patterns in the same cluster
b(i)	the smallest average distance between the frequent pattern *i* and the frequent patterns in the other clusters, of which *i* is not part
$centroid\left(a_{i}\right)$	Activity corresponding to clusters’ centroids
dailyRoutine	Routine for a day
dailyRoutine	activity sequence representing the baseline
dailyTimespanVectors	timespan of activities for all days and all periods of the dataset
optional variable	Specifies if an activity is mandatory or not.
period	Specific day period. It can be night, morning, afternoon, or evening.
s(i)	Silhouette score
$\delta \left(X_{i},\;X_{j}\right)$	distance between the cluster $X_{i}$, and the cluster $X_{j}$

**Table 2:** Comparison of research literature approaches.

Ref.	Method	ADL Features	Flexibility	Routine Definition	Solution Features
[16]	DBSCAN algorithm	Activity start time, duration	Not considered	Sequence of activities	Single routine, deviations are not considered
[17]	FMR–AD algorithm & fuzzy rules	Activity frequency, regularity	Not considered	Sequence of activities and their frequency	Single routine, deviations detection
[14]	Partition Around Medoids algorithm	Activity label	Not considered	Sequence of activities	A single rigid routine
[18]	Fourier series representation combined with K-means	Activity label, activity duration	Activities time-related variability	Sequence of activities and associated durations	Three routines correspond to morning, afternoon, and night.
[19]	Weighted kernel k-means algorithm & nominal matrix factorization method	Activity label	Not considered	Sequence of activities	A set of routines for the monitoring period
[27]	Hidden Markov models combined with Baum–Welch algorithm and Viterbi algorithm	Activity label, location, posture of the person, activity duration	Not considered	Sequence of activities, associated durations, and locations	A single routine
[28]	Markov Decision Process combined with relative entropy inverse reinforcement learning	Sensor’s locations, sensors activation time	Not considered	Trajectory vector extracted based on the sensor’s locations and sensors activation time	A single rigid routine represented as a trajectory vector
[26]	Transition probability matrix	Time spent by a person in a particular room	Not allowed	State transition model where states are the home’s rooms, and the connections are the transitions	Normal mobility behavior of a person and deviations from it
[30]	Grey model with a Markovian model	Activity frequency, activity duration	Variability related to the duration and frequency of activities	Sequence of activities, associated durations, and frequency	A single routine
[29]	Probabilistic spatio-temporal model combined with K-means	The location of the subject and the sensor activation timespan	Not allowed	Sequence of location events, start time, end time, and location label	Two rigid behavioral patterns; Normal and deviations from it
[31]	Shapiro-Wilk test combined with a non-parametrical statistical method	Time duration and frequency of visiting the rooms	Variability related to the duration	Activity’s duration and transitions between the rooms	Single routine
Our solution	Deep learning, GAP-BIDE algorithm, collaborative clustering	ADL timespan, start time, end time	Variability related to Activities time, sequence gaps variability	Sequence of four sub-routines corresponding to each period of the day	A single flexible routine composed of mandatory activities, optional activities, alternative variants of activities

**Table 3:** Day division in periods.

Period	Time
Night	00:00:00 PM–06:59:59 AM
Morning	07:00:00 AM–11:59:59 AM
afternoon	12:00:00 AM–6:59:59 PM
Evening	7:00:00 PM–11:59:59 PM

**Table 4:** Dunn index and Davies-Bouldin index values for Agglomerative Hierarchical Clustering.

Number of Clusters	Dunn Index	Davies-Bouldin Index	Distribution of Elements in Clusters
**2**	0.49747	0.50333	Cluster 0: 11 elements; Cluster 1: 3 elements
**3**	0.75388	0.47348	Cluster 0: 3 elements; Cluster 1: 8 elements Cluster 2: 3 elements
**4**	0.76397	0.37467	Cluster 0: 8 elements; Cluster 1: 2 elements Cluster 2: 3 elements; Cluster 3: 1 element
**5**	0.46472	0.457	Cluster 0: 3 elements; Cluster 1: 4 elements Cluster 2: 4 elements; Cluster 3: 1 element Cluster 4: 2 elements
**6**	0.46472	0.36311	Cluster 0: 4 elements; Cluster 1: 4 elements Cluster 2: 2 elements; Cluster 3: 1 element Cluster 4: 2 elements; Cluster 5: 1 element

**Table 5:** Coverage Metric Values.

Clustering Configuration	Routine Coverage Value
Collaborative Clustering (Union)	89.63%
K-Means	77.44%
Agglomerative Clustering	88.41%

**Table 6:** Number of clusters and clustering execution time variation.

Nr Clusters	Execution Time (s)	
	Agglomerative Clustering	K-Means
2	0.01	1.49
3	0.02	1.17
4	0.02	1.00
5	0.03	1.20
6	0.02	1.42

**Table 7:** Data size impact on clustering execution time.

Data Set Size (Days)	Execution Time (s)
400	1.19
300	0.62
200	0.23

**Table 8:** Parameters effect on deep learning models performance.

Parameters Varied			Average Values		
Window Size	Stride	Number of Models	Accuracy	Loss	Learning Rate
200	50	15	0.945	0.143	0.003
200	50	15	0.936	0.164	0.025
600	30	30	0.977	0.059	0.015
600	35	30	0.979	0.054	0.012
600	40	10	0.978	0.059	0.015
600	50	40	0.976	0.063	0.011
900	30	35	0.978	0.056	0.008
